# A importância de reconhecer a síndrome antifosfolípide na medicina vascular

**DOI:** 10.1590/1677-5449.011416

**Published:** 2017

**Authors:** Andreas Funke, Adriana Danowski, Danieli Castro Oliveira de Andrade, Jozelia Rêgo, Roger Abramino Levy

**Affiliations:** 1 Universidade Federal do Paraná – UFPR, Hospital de Clínicas, Curitiba, PR, Brasil.; 2 Hospital Federal dos Servidores do Estado – HFSE, Rio de Janeiro, RJ, Brasil.; 3 Universidade de São Paulo – USP, Faculdade de Medicina, São Paulo, SP, Brasil.; 4 Universidade Federal de Goiás – UFG, Faculdade de Medicina, Goiânia, GO, Brasil.; 5 Universidade do Estado do Rio de Janeiro – UERJ, Rio de Janeiro, RJ, Brasil.

**Keywords:** síndrome antifosfolípide, anticoagulante do lúpus, anticorpos anticardiolipina, trombose, autoimunidade

## Abstract

A síndrome antifosfolipíde (SAF) é uma doença autoimune sistêmica caracterizada por trombose arterial ou venosa recorrente e/ou morbidade gestacional e pela presença dos anticorpos antifosfolipídeos, podendo apresentar outras manifestações vasculares, como microangiopatia, arteriopatia crônica e SAF catastrófica. Determinados testes laboratoriais para a síndrome (por exemplo, o anticoagulante lúpico) podem sofrer interferência do uso de medicações anticoagulantes, dificultando o diagnóstico. A fisiopatologia da SAF é complexa, sendo enumerados no texto diversos mecanismos patogênicos relacionados à coagulação, ao endotélio e às plaquetas. Por fim, discutimos o tratamento da SAF de acordo com a presença e o tipo de manifestações clínicas, o uso dos anticoagulantes orais diretos e o manejo perioperatório de pacientes com SAF.

## INTRODUÇÃO

A síndrome antifosfolípide (SAF) é uma doença autoimune sistêmica caracterizada por trombose arterial ou venosa recorrente e/ou morbidade gestacional acompanhadas pela presença persistente dos assim chamados “anticorpos antifosfolipídeos”^1^ (aPL), detectados através dos exames laboratoriais para anticoagulante lúpico (*lupic anticoagulant*, LA), anticardiolipina (aCL) IgG e IgM, e anti-β2-glicoproteína I (anti-β2-GPI) IgG e IgM. A SAF pode ocorrer em associação com outra doença autoimune, mais frequentemente o lúpus eritematoso sistêmico (LES), ou ser observada de forma isolada (SAF primária)^1^^,^ 2.

## MANIFESTAÇÕES CLÍNICAS

Qualquer órgão ou sistema pode ser afetado pela SAF. A síndrome manifesta-se mais comumente por trombose venosa profunda (TVP). Suas manifestações, porém, não se limitam ao leito venoso, podendo também manifestar-se através de trombose arterial (com ou sem aterosclerose subjacente), resultando, por exemplo, num acidente vascular encefálico (AVE) isquêmico, ou ainda cursar com microangiopatia trombótica, tal como a observada na nefropatia da SAF^3^. Também tem sido atribuída à SAF a ocorrência de vasculopatia associada a hiperplasia intimal acentuada e estenose arterial^3^^,^ 4. Por fim, um quadro acelerado de aterosclerose tem sido descrito em pacientes com aPL ou SAF^5^^-^ 7. A frequência das diversas manifestações da SAF, de acordo com a análise de uma grande coorte (1.000 pacientes) europeia^8^^,^ 9, pode ser vista na Tabela 1.

**Tabela 1:** Manifestações clínicas da SAF.

Frequentes (> 20% dos casos):	
Tromboembolismo venoso	Trombocitopenia
Abortamento ou perda fetal	AVE/AIT
Migrânea refratária	Livedo reticular
Menos frequentes (10-20% dos casos):	
Doença valvar cardíaca	Pré-eclâmpsia ou eclâmpsia
Nascimento prematuro	Anemia hemolítica
Doença arterial coronariana	
Incomuns (< 10% dos casos):	
Epilepsia	Demência vascular
Coreia	Trombose de veia ou artéria retiniana
Amaurose fugaz	Hipertensão pulmonar
Úlceras de perna	Gangrena digital
Osteonecrose	Microangiopatia renal
Isquemia mesentérica	
Raras (< 1% dos casos):	
Hemorragia suprarrenal	Mielite transversa
Síndrome de Budd-Chiari	CAPS

AVE: acidente vascular encefálico; AIT: ataque isquêmico transitório; CAPS: SAF catastrófica. Adaptado de Ruiz-Irastorza et al.^9^.

Raramente, observa-se um quadro devastador e de rápida instalação conhecido como SAF catastrófica (*catastrophic antiphospholipid syndrome*, CAPS), caracterizado por múltiplas oclusões vasculares (em mais de três órgãos ou sistemas) num curto período de tempo (menos de 1 semana). Mais da metade dos casos de CAPS ocorre na presença de fatores desencadeantes (gatilhos) identificáveis, tais como infecções bacterianas ou virais, procedimentos cirúrgicos, descontinuação do tratamento anticoagulante, complicações obstétricas, neoplasia e LES concomitante. A CAPS está associada a elevadas taxas de mortalidade, variando de 36,8% a mais de 50% dos casos, conforme diferentes publicações^10^^,^ 11.

## DIAGNÓSTICO

Os critérios preliminares de classificação publicados em 1999 (Sapporo) e atualizados em 2006 (Sydney)^12^ definem como SAF a presença de pelo menos um critério clínico e um critério laboratorial, conforme especificado na Tabela 2. Esses critérios têm por objetivo a definição das características da síndrome para fins de estudos etiológicos e terapêuticos. Eles também são frequentemente utilizados para diagnóstico preciso da SAF na prática clínica, mas não devem ser considerados requisitos absolutos para a indicação ou não de tratamento.

**Tabela 2:** Critérios atualizados (Sydney) para classificação da SAF.

Critérios clínicos	1. Trombose venosa^*^, arterial ou de pequenos vasos Confirmada objetivamente (imagem ou histopatologia^**^)
	2. Morbidade gestacional
	(a) uma ou mais ocorrências inexplicadas de morte de feto morfologicamente normal na ou após a 10^a^ semana de gestação, confirmada por ultrassom ou exame do feto; ou
	(b) um ou mais nascimentos prematuros (antes da 34^a^ semana de gestação) de neonato morfologicamente normal devido a eclâmpsia, pré-eclampsia severa ou insuficiência placentária; ou
	(c) três ou mais abortamentos espontâneos inexplicados consecutivos antes da 10^a^ semana de gravidez, excluídas causas anatômicas ou hormonais maternas e causas cromossômicas paternas e maternas.
Critérios laboratoriais	(a) anticoagulante lúpico (LA) detectado conforme as diretrizes da ISTH^§^ em duas ou mais ocasiões separadas por pelo menos 12 semanas; ou
	(b) anticorpo anticardiolipina (aCL) IgG ou IgM detectado no soro ou no plasma em título médio a alto (> 40 unidades GPL ou MPL ou > 99^o^ percentil) em duas ou mais ocasiões separadas por no mínimo 12 semanas, mensurado por ELISA padronizado; ou
	(c) anticorpo anti-β2-glicoproteína I (anti-β2-GPI) detectado no soro ou no plasma em duas ou mais ocasiões separadas por pelo menos 12 semanas, mensurado através de ELISA padronizado

* Não inclui trombose venosa superficial;

** Sem inflamação significativa na parede vascular (vasculite)

§ Sociedade Internacional de Trombose e Hemostasia. Adaptado de Miyakis et al.^12^.

Apenas as manifestações clínicas e achados laboratoriais mais específicos para a SAF foram incluídos nos critérios de classificação. Outros achados clínicos e laboratoriais também associados à SAF são denominados manifestações ou exames “não critério”. Entre as manifestações “não critério” da SAF podem ser citadas a doença valvar cardíaca, o livedo reticular, as úlceras cutâneas, a trombocitopenia, a nefropatia da SAF e as manifestações neurológicas relacionadas aos aPL^12^^,^ 13. Testes laboratoriais não-critério incluem: anticorpos aCL IgA, anti-β2-GPI IgA, anti-fosfatidilserina (aPS), anti-fosfatidiletanolamina (aPE), anti-protrombina isolada (aPT-A), anti-complexo fosfatidilserina-protrombina (aPS/PT) e anti-domínio I da β2-glicoproteína I^12^^,^ 14.

### Efeito da terapia anticoagulante na investigação laboratorial da SAF

Na prática, pode ser necessário realizar-se a investigação laboratorial da SAF em pacientes que já estejam sendo tratados com medicações anticoagulantes parenterais ou orais. Nesse contexto, não se espera influência significativa sobre os resultados dos testes de ELISA aCL e anti-β2-GPI, mas pode haver dificuldades no diagnóstico correto do LA.

As diretrizes da *International Society on Thrombosis and Haemostasis* (ISTH)^15^ recomendam realizar-se o exame de LA de 1 a 2 semanas após a descontinuação do tratamento com antagonistas da vitamina K (AVK) ou quando a razão normalizada internacional (RNI) for menor que 1,5. Pode-se utilizar uma ponte terapêutica de heparina de baixo peso molecular (HBPM) quando da descontinuação dos AVK, cuidando-se para que a última administração de heparina tenha ocorrido mais de 12 horas antes da coleta da amostra para o LA. Uma consideração alternativa é a realização dos exames para LA na vigência do tratamento com AVK, num momento em que a RNI esteja entre 1,5 e < 3,0 e fazendo-se a diluição prévia da amostra de plasma do paciente na proporção de 1:1 com pool de plasma normal. Nesse caso, um LA em título baixo pode não ser detectado, já que a amostra é diluída pela metade.

Um fato a ser destacado, diante da crescente popularidade dos novos anticoagulantes orais diretos (AODs), tais como a rivaroxabana e o etexilato de dabigatrana, no tratamento de pacientes com tromboembolismo venoso, é a ocorrência de resultados falso-positivos nos testes usuais para o LA [tempo de veneno de víbora de Russel diluído (*dilute Russell's viper venom time*, dRVVT) e tempo de tromboplastina parcial ativada (TTPA)] em vigência de concentrações terapêuticas dessas medicações^16^^,^ 17. No caso específico da rivaroxabana, esse efeito pode ser evitado utilizando-se o tempo de veneno da cobra Taipan ou tempo de ecarina para diagnóstico do LA^18^, ou aguardando-se pelo menos 24 horas após a última dose dessa medicação para pesquisa do LA através das técnicas usuais^19^.

## FISIOPATOLOGIA DA SAF

A presença de aPL, especialmente a presença do LA mediado por anticorpos anti-β2-GPI, confere risco aumentado de trombose e/ou morbidade gestacional. A infusão experimental de autoanticorpos oriundos de pacientes com SAF potencializa a formação de trombos em camundongos nos quais tinha sido previamente induzida uma lesão vascular. Entretanto, o mesmo efeito não ocorre na ausência de uma lesão vascular prévia, sugerindo que a ocorrência da SAF se dá através de duas etapas distintas (hipótese *double-hit*): (1) existência de um estado pró-trombótico latente, induzido pelos autoanticorpos circulantes; (2) lesão ou ativação endotelial (gatilho) desencadeando o evento trombótico. Estudos recentes indicando níveis plasmáticos aumentados de micropartículas circulantes derivadas de plaquetas e de células endoteliais em pacientes com SAF ou aPL corroboram o conceito de um contínuo estado pró-trombótico nesses pacientes^20^^,^ 21.

Potenciais mecanismos envolvidos na patogênese da SAF incluem^22^:

- Imunogenicidade aumentada do domínio I da β2-glicoproteína I plasmática, decorrente do estresse oxidativo;
- Mudança conformacional da molécula de β2-glicoproteína I ao ligar-se aos fosfolipídeos aniônicos da membrana celular, possibilitando a ligação e efeitos patogênicos subsequentes dos autoanticorpos;
- Interferência desses autoanticorpos na função da sintetase endotelial de óxido nítrico;
- Indução pelos aPL do aumento da expressão de fator tecidual em células endoteliais e monócitos;
- Ativação descontrolada do fator XI e consequente predisposição para trombose venosa e arterial;
- Potencialização da ativação plaquetária pela interação entre β2-glicoproteína I, receptores plaquetários e anticorpos anti-β2-GPI;
- Interferência dos complexos antígeno/anticorpo β2-GPI/anti-β2-GPI na função protetora da anexina A5 contra a ativação da coagulação;
- Ativação de complemento (C3 e C5) mediada por anticorpo, resultando em perda fetal e/ou trombose;
- Aumento da expressão dos receptores do tipo Toll 7 e 8 (TLR7 e TLR8); e
- Em pacientes com nefropatia da SAF, ativação pelos aPL da via do complexo do alvo da rapamicina em mamíferos (*mammalian target of rapamycin complex*, mTORC), induzindo acentuada hiperplasia intimal e vasculopatia^23^.

## TRATAMENTO SAF – MORBIDADE GESTACIONAL

O atendimento de pacientes com SAF obstétrica inclui medidas não farmacológicas, tais como o acompanhamento por equipe multidisciplinar em clínica de gestação de alto risco e o rigoroso controle clínico e laboratorial, bem como o monitoramento ecográfico do crescimento fetal e da circulação uteroplacentária^24^^,^ 25. Pacientes com história de SAF com manifestações unicamente obstétricas podem ser tratadas, no caso de nova gestação, com aspirina em dose baixa (ADB) (75-100 mg/dia) associada a heparina não fracionada (HNF) ou HBPM em doses profiláticas até 6 semanas após o parto^24^. Entretanto, gestantes com SAF e história de eventos trombóticos requerem terapia antitrombótica durante toda a gravidez e no puerpério, com a combinação de ADB e dose anticoagulante plena de heparina (HNF ou HBPM)^24^^,^ 26.

Após o término do ciclo gravídico-puerperal, pacientes que apresentaram SAF gestacional, mesmo sem antecedentes de trombose, permanecem sob risco aumentado de eventos trombóticos, recomendando-se, nessas circunstâncias, além do controle dos fatores de risco clássicos para trombose, a profilaxia primária com ADB por prazo indeterminado^26^.

## TRATAMENTO DA SAF – MANIFESTAÇÕES TROMBÓTICAS

### Estratificação do risco de trombose e de hemorragia

Qualquer decisão terapêutica em pacientes com SAF deve levar em consideração o risco de recorrência de trombose e os riscos decorrentes do tratamento anticoagulante (por exemplo, hemorragia)^27^. Recomenda-se de forma geral a estratificação do risco de trombose através da identificação dos fatores de risco cardiovascular (principalmente tabagismo, hipertensão, diabetes, dislipidemia, e obesidade), da presença ou não de doença autoimune associada (por exemplo, LES) e da configuração ou não de um perfil de aPL de alto risco^27^, que inclui (1) positividade do LA; (2) positividade tripla, isto é, presença concomitante de LA, aCL e anti-b2-GPI; (3) Níveis altos e persistentes de aCL em pacientes com LES. Fatores que podem aumentar o risco de complicações hemorrágicas do tratamento anticoagulante incluem o uso associado de aspirina (especialmente se > 100 mg/dia), idade > 75 anos, hemorragia grave prévia, polifarmácia, câncer e alterações de substância branca cerebral (leucoaraiose)^27^.

### Tromboprofilaxia secundária

Pacientes que sofreram um ou mais eventos trombóticos e que preenchem os critérios de classificação da SAF apresentam risco aumentado de recorrência desse tipo de eventos, sendo indicada terapia anticoagulante a longo prazo. No caso de trombose venosa prévia, indica-se o uso de AVK com uma meta de RNI de 2,0 a 3,0. Para pacientes com SAF e trombose arterial, a recomendação prevalente^1^^,^ 26 ^,^ 27, baseada na opinião de especialistas, é o tratamento a longo prazo com AVK e uma meta de RNI >3,0 (regime intensivo), ou RNI de 2,0 a 3,0 em associação com antiagregação plaquetária (ADB). O tratamento de pacientes com eventos trombóticos mas que não preenchem critérios de classificação para SAF, isto é, que têm apenas positividade em baixos títulos e/ou não persistente dos aPL, não deve ser distinto daquele a ser indicado se esses exames fossem negativos^27^.

A duração da tromboprofilaxia secundária, no caso de SAF com trombose arterial ou venosa confirmada pelos critérios de classificação (Tabela 2), é por prazo indeterminado. Porém, no caso de um primeiro evento de trombose venosa que tenha um fator precipitante transitório identificado e um perfil de baixo risco de aPL (positividade isolada, não persistente e em títulos baixos de aCL ou anti-β2-GPI, portanto critérios insuficientes para SAF), pode ser considerada a descontinuação do tratamento anticoagulante após 3 a 6 meses, decisão essa que pode ser eventualmente subsidiada por exames de eco-Doppler e dímero-D visando excluir trombose venosa residual significativa.

### Tromboprofilaxia primária

A tromboprofilaxia primária de longo prazo em pacientes portadores de aPL mas sem trombose prévia é um tema controverso. Um estudo prospectivo não indicou benefício do uso da ADB^28^, porém uma metanálise de 11 estudos detectou uma ação protetora dessa intervenção contra eventos trombóticos (arteriais mas não venosos), e embora essa proteção tenha desaparecido quando a análise foi restrita a apenas estudos prospectivos e de melhor qualidade^1^^,^ 29, outra metanálise que usou dados individuais de pacientes de cinco coortes voltou a encontrar evidência de proteção^30^.

As seguintes recomendações da força-tarefa conduzida durante o 13^o^ Congresso Internacional de Anticorpos Antifosfolipídeos^27^ permanecem válidas quanto à abordagem de pacientes com aPL mas sem antecedentes de trombose: (1) avaliação e abordagem dos fatores de risco cardiovascular associados; (2) uso de HBPM ou equivalente durante períodos de risco aumentado de eventos tromboembólicos (cirurgia, imobilização, puerpério); (3) prescrição de antiagregante plaquetário (ADB) para pacientes de maior risco, tais como pacientes com fatores de risco pró-trombóticos concomitantes e pacientes com perfil de alto risco de aPL (ver acima); e (4) uso de antiagregante plaquetário (ADB) e hidroxicloroquina em pacientes com LES e que sejam positivos para o LA ou possuam níveis altos e persistentes de aCL.

### Anticoagulantes orais diretos não inibidores de vitamina K

A tromboprofilaxia secundária a longo prazo com varfarina ou outros AVK apresenta vários inconvenientes, tais como a necessidade de monitoramento regular do RNI, a existência de múltiplas interações medicamentosas e alimentares e, em alguns casos, dificuldade em se atingir ou manter níveis adequados e estáveis de anticoagulação. Outro problema levantado na literatura é a interação variável entre o LA e os reagentes utilizados na avaliação do tempo de ativação de protrombina (TAP), por vezes resultando em valores basais prolongados e menor confiabilidade dos valores de RNI no monitoramento da intensidade da anticoagulação^16^^,^ 31.

AODs, incluindo um inibidor direto da trombina (etexilato de dabigatrana), e dois inibidores diretos do fator Xa (rivaroxaba e apixabana), estão disponíveis comercialmente em nosso país, possuindo como indicações aprovadas, entre outras, a profilaxia de TVP e/ou tromboembolismo venoso. A eficácia e segurança do seu emprego para essas indicações foram confirmadas por pelo menos três revisões sistemáticas^32^^-^ 34. Essas medicações apresentam como vantagens com relação ao uso de AVK: administração em doses fixas, geralmente sem a necessidade de monitoramento laboratorial da sua ação anticoagulante; a ausência de interações significativas com álcool ou alimentos; e um menor número de interações medicamentosas relevantes^16^^,^ 17.

Diante das inconveniências no uso dos AVK e das vantagens trazidas pelos AODs, é natural que exista o interesse de utilizar esses medicamentos mais novos também no tratamento da SAF. Estima-se que quase 10% dos pacientes com TVP sejam portadores de SAF^35^, do que se depreende que provavelmente houve pacientes com SAF incluídos nos estudos dos AODs. Entretanto, considerando-se não ter havido documentação sistemática da positividade de aPL naqueles estudos, seus resultados podem não ser generalizáveis a pacientes com SAF^36^. Além disso, a intervenção comparada naqueles mesmos estudos foi a varfarina com alvo de RNI de 2,5 (2,0-3,0), geralmente apropriada para a profilaxia secundária de trombose venosa, mas podendo representar um nível de proteção insuficiente para pacientes com SAF, particularmente aqueles com trombose arterial. Há atualmente, segundo o conhecimento dos autores, pelo menos dois estudos clínicos em andamento avaliando o uso de rivaroxabana em pacientes com SAF: o estudo *Rivaroxaban In Antiphospholipid Syndrome* (RAPS)^36^, o qual vem recrutando pacientes com SAF e antecedentes de trombose venosa, e o estudo *Rivaroxaban in Thrombotic Antiphospholipid Syndrome* (TRAPS)^37^, cuja população estudada é a de pacientes com SAF e trombose venosa, arterial ou microvascular, e perfil de positividade tripla de aPL. Os resultados desses estudos, quando disponíveis, poderão esclarecer melhor o papel dos AODs no tratamento de pacientes com SAF.

Se por um lado os resultados de estudos clínicos específicos ainda não estão disponíveis, podem-se encontrar na literatura diversos relatos de casos e/ou de séries de casos do uso de AODs em pacientes com SAF, trazendo experiências contraditórias. Numa série^16^ de 35 pacientes com SAF e antecedentes de trombose venosa com indicação de anticoagulação em intensidade moderada (RNI 2 a 3), mas com dificuldade de atingir ou manter a meta terapêutica, efetuou-se a troca dos AVK por rivaroxabana 20 mg ao dia, não se observando novas ocorrências de tromboembolismo venoso durante um seguimento mediano de 10 (6-24) meses. Em outra publicação^38^, relata-se o tratamento de 26 pacientes com SAF e antecedentes de trombose venosa e/ou arterial prévia com dabigatrana ou rivaroxabana. Após um seguimento mediano de 19 (8-29) meses, ocorreu descontinuação do tratamento em apenas quatro casos (trombose recorrente em um paciente, complicações hemorrágicas em duas pacientes e recorrência de migrânea em uma paciente). Entretanto, também encontramos na literatura relatos de experiências bastante desfavoráveis do uso de AODs em pacientes com SAF. Schaefer et al.^39^ reportaram três casos (dois de SAF primária com eventos arteriais e um de SAF e LES com eventos venosos) que apresentaram novos eventos trombóticos após a troca da varfarina por um AOD (dabigatrana ou rivaroxabana). Win & Rodgers relatam também três casos de SAF de difícil tratamento que apresentaram novos eventos trombóticos ou isquêmicos em vigência do uso de rivaroxabana ou dabigatrana^40^. Em outra série de casos, relatada por Signorelli et al.^41^, oito pacientes com SAF e história prévia de tromboses venosas e/ou arteriais (três deles com tripla positividade de aPL) desenvolveram novos eventos trombóticos durante tratamento com rivaroxabana, reportando-se o reinício do tratamento com AVK em sete desses pacientes, sem nova recidiva de trombose desde então.

As recomendações da força-tarefa de tendências terapêuticas na síndrome antifosfolípide, conduzida durante o 14^o^ Congresso Internacional sobre Anticorpos Antifosfolipídeos^42^, são de que os AVK continuem sendo a base da anticoagulação em pacientes com SAF trombótica, podendo se considerar o uso de AODs em pacientes com tromboembolismo venoso recorrente ou inicial que ocorra na ausência ou sob níveis subterapêuticos de anticoagulação e apenas se houver alergia ou intolerância conhecida aos AVK ou controle anticoagulante inadequado. Conforme as mesmas recomendações, não há dados que recomendem o uso dos AODs em pacientes com SAF e tromboembolismo venoso recorrente observado sob níveis terapêuticos de anticoagulação ou que apresentem trombose arterial pela SAF. Além disso, essas recomendações alertam para o papel crucial da aderência dos pacientes ao tratamento com AODs, uma vez que seu efeito anticoagulante não é usualmente monitorado, não sendo portanto a má aderência do paciente ao uso de AVK justificativa para a indicação de um AOD. Por fim, advertem quanto à inexistência de antídotos para os AODs no caso de complicações hemorrágicas graves ou necessidade urgente de cirurgia.

Um resumo do tratamento da SAF de acordo com o tipo de manifestação (gestacional ou trombótica) pode ser encontrado na Tabela 3. As tromboprofilaxias primária e secundária da SAF estão sumarizadas na Tabela 4. 

**Tabela 3:** Resumo do tratamento da SAF.

SAF com morbidade gestacional	
Medidas gerais	Equipe multidisciplinar. Monitoramento clínico/laboratorial/ecográfico
Farmacoterapia	Sem trombose prévia: ADB + dose profilática de HNF ou HBPM Com trombose prévia: ADB + dose terapêutica de HNF ou HBPM Após o parto/puerpério: ADB
SAF com Manifestações trombóticas	
Medidas gerais	Estratificação de risco para trombose e/ou hemorragia
Farmacoterapia	Tromboprofilaxia secundária

**Tabela 4:** Tromboprofilaxia na SAF.

Tromboprofilaxia secundária (SAF confirmada)	
Trombose venosa	Anticoagulação com AVK, RNI 2,0 a 3,0
Trombose arterial	Anticoagulação com AVK, RNI > 3,0 ou Anticoagulação com AVK, RNI 2,0 a 3,0 + ADB
Tromboprofilaxia primária (positividade de aPL, ausência de critérios clínicos)	
Geral	Tromboprofilaxia com HBPM ou equivalente em períodos de alto risco (cirurgia, imobilização, puerpério)
Com fatores de risco associados (risco Cardiovascular, perfil de aPL de alto risco)	ADB
Pacientes com LES e positividade para o LA ou níveis altos/persistentes de aCL	ADB + hidroxicloroquina

### Tratamento da SAF catastrófica (CAPS)

Pela sua gravidade, a CAPS requer abordagem imediata e vigorosa. Fatores precipitantes reversíveis (por exemplo, infecções) devem ser identificados e tratados. Análises de séries e relatos de casos^10^^,^ 11 ^,^ 43 ^-^ 45 indicam maior sobrevida com o uso de terapia combinada tríplice que inclui a anticoagulação plena com heparina e a corticoterapia em doses altas associadas à plasmaférese e/ou imunoglobulina intravenosa (IgIV), sendo esta última preferencial se houver infecção ativa. Caso se use ambas (plasmaférese e IgIV), a sequência de plasmaféreses deverá ser concluída previamente ao início da administração de IgIV. Na presença de LES ou outra doença autoimune subjacente à CAPS, tratamento adicional com ciclofosfamida ou outro imunossupressor poderá ser indicado. O uso de rituximabe tem sido aventado no tratamento de CAPS refratária, recidivante, na presença de anemia hemolítica microangiopática ou quando a anticoagulação for contraindicada devido a complicações hemorrágicas concomitantes^11^.

### Manejo perioperatório de pacientes com aPL ou SAF^46^

A realização de um procedimento cirúrgico em pacientes com aPL ou SAF pode precipitar a ocorrência de eventos trombóticos^47^ e mesmo de CAPS^11^. É portanto necessário um planejamento pré-operatório adequado visando a sua profilaxia. A equipe médica (clínico, cirurgião, anestesiologista) deverá, de comum acordo, definir a abordagem referente à cronologia da suspensão pré-operatória dos AVK, uso da ponte de HBPM, aplicação de dispositivos de compressão intermitente, deambulação pós-operatória precoce, reintrodução pós-operatória dos AVK e estratégia de monitoramento laboratorial^46^. Essa abordagem deverá levar em consideração a história de eventos trombóticos ou hemorrágicos, a existência de um perfil clinicamente relevante de aPL (título > 40 unidades GPL ou MPL, persistência > 12 semanas, e a detecção do LA feita de acordo com as diretrizes da ISTH) e a presença de outros fatores de risco para trombose ou hemorragia^46^^,^ 48. Embora 20 a 40% dos pacientes com SAF apresentem trombocitopenia, mais comumente acima de 70.000 plaquetas/mm^3^, esta raramente resulta em hemorragia significativa e não reduz o risco de trombose^49^. Na ausência de terapia anticoagulante, um TTPA aumentado (acima do valor de referência) pode refletir a presença do LA e um TAP minimamente prolongado (acima do limite superior de normalidade) pode indicar a presença de anticorpos antiprotrombina. Alterações mais acentuadas do TAP (RNI > 2,0) requerem investigação de outras causas incluindo a hipoprotrombinemia associada ao LA^46^^,^ 50.

A prevenção da trombose perioperatória em pacientes com SAF inclui medidas gerais, tais como (i) minimizar a manipulação intravascular de acesso e monitoramento; (ii) reduzir a estase venosa, evitando o uso de torniquetes na coleta de sangue e reduzindo a frequência de insuflação dos manguitos de pressão arterial; (iii) tromboprofilaxia, combinando modalidades mecânicas (meias de compressão gradual ou dispositivos de compressão pneumática intermitente) e medidas farmacológicas^46^. A tromboprofilaxia farmacológica perioperatória em pacientes com SAF sob anticoagulação oral com AVK é feita de forma parenteral com o uso de uma ponte com HBPM (enoxaparina) ou HNF, transicionando-se assim que possível para os AVK no pós-operatório (a HNF ou HBPM só deverá ser suspensa quando a RNI estiver no alvo)^46^^,^ 51. A ocorrência de complicações hemorrágicas não elimina o risco de tromboembolismo, sendo recomendado nessa situação o uso intensivo das modalidades mecânicas e, assim que possível, o reinício das medidas farmacológicas de tromboprofilaxia^46^.

## CONSIDERAÇÕES FINAIS

A SAF apresenta grande importância para a medicina vascular, por ser uma causa reconhecida de trombose venosa e/ou arterial recorrentes. Seu diagnóstico depende do reconhecimento de suas manifestações clínicas trombóticas e/ou gestacionais e da solicitação e interpretação adequada de testes laboratoriais para a síndrome. O resultado do exame para o LA pode modificar-se pelo uso de medicações anticoagulantes parenterais ou orais, sendo recomendado um cuidado especial na seleção do momento da coleta da amostra e na sua interpretação nessas circunstâncias. Um crescente número de mecanismos patogênicos da SAF vem sendo desvendado, o que pode viabilizar o desenvolvimento de opções terapêuticas novas que não envolvam o uso de anticoagulantes. A adequada abordagem terapêutica desses pacientes deve levar em consideração a história de eventos trombóticos e/ou morbidade gestacional prévia e outros fatores de risco cardiovascular associados. Embora os AODs tenham vários benefícios frente ao uso de varfarina ou de outros AVK no tratamento e profilaxia da trombose venosa na população geral, sua eficácia na SAF ainda aguarda confirmação em estudos específicos. O manejo perioperatório de pacientes com SAF requer cuidados especiais voltados à profilaxia tanto de manifestações tromboembólicas quanto de complicações hemorrágicas.
