# Supplementary material for: A importância de reconhecer a síndrome antifosfolípide na medicina vascular
Source: J Vasc Bras. 2017 Apr-Jun;16(2):140–9. [Article in Portuguese] doi: 10.1590/1677-5449.011416 (PMC5915862; doi:10.1590/1677-5449.011416)
Supplement: Translation

# Translation: The importance of recognizing antiphospholipid syndrome in vascular medicine

**Authors:** Andreas Funke, Adriana Danowski, Danieli Castro Oliveira de Andrade, Jozelia Rêgo, Roger Abramino Levy

**Affiliations:** 1 Universidade Federal do Paraná – UFPR, Hospital de Clínicas, Curitiba, PR, Brazil.; 2 Hospital Federal dos Servidores do Estado – HFSE, Rio de Janeiro, RJ, Brazil.; 3 Universidade de São Paulo – USP, Faculdade de Medicina, São Paulo, SP, Brazil.; 4 Universidade Federal de Goiás – UFG, Faculdade de Medicina, Goiânia, GO, Brazil.; 5 Universidade do Estado do Rio de Janeiro – UERJ, Rio de Janeiro, RJ, Brazil.

**Keywords:** antiphospholipid syndrome, lupus anticoagulant, anticardiolipin antibodies, thrombosis, autoimmunity

## Abstract

Antiphospholipid syndrome (APS) is a systemic autoimmune disease characterized by recurrent arterial or venous thrombosis and/or gestational morbidity and by the presence of antiphospholipid antibodies. It can also cause other vascular manifestations such as microangiopathy, chronic arteriopathy and catastrophic APS (CAPS). Certain laboratory tests for the syndrome (for example, the lupus anticoagulant test) can be affected by the use of anticoagulant agents, making diagnosis more difficult. The pathophysiology of APS is complex, and several mechanisms of pathogenesis related to coagulation, endothelium, and platelets are discussed in this article. We conclude by discussing treatment of APS according to the presence and type of clinical manifestations, use of direct oral anticoagulants (DOAs), and perioperative management of patients with APS.

## INTRODUCTION

Antiphospholipid syndrome (APS) is a systemic autoimmune disease characterized by recurrent arterial or venous thrombosis and/or gestational morbidity and persistent presence of what have become known as “antiphospholipid antibodies”^1^ (aPL), which can be detected by laboratory tests for lupus anticoagulant (LA), anticardiolipin (aCL) IgG and IgM, and anti-β2-glycoprotein I (anti-β2-GPI) IgG and IgM. Antiphospholipid syndrome can occur in combination with other autoimmune diseases, most commonly systemic lupus erythematosus (SLE), and it can also be found in isolation (primary APS).^1^^,^ 2

## CLINICAL MANIFESTATIONS

Antiphospholipid syndrome can affect any organ or system. It most often manifests through deep venous thrombosis (DVT). However, its manifestations are not limited to the venous bed and it can also manifest through arterial thrombosis (with or without subjacent atherosclerosis), resulting, for example, in an ischemic stroke, or thrombotic microangiopathy, as is seen in nephropathy of APS.^3^ Vascular disease associated with accentuated intimal hyperplasia and arterial stenosis have also been attributed to APS.^3^^,^ 4 Finally, cases of accelerated atherosclerosis have also been described in patients with aPL or APS.^5^^-^ 7 Table 1 lists the frequencies of the various different manifestations of APS, according to analysis of a large European cohort (1,000 patients.^8^^,^ 9

**Table 1:** Clinical manifestations of APS.

Frequent (> 20% of cases):	
Venous thromboembolism	Thrombocytopenia
Abortion or loss of fetus	Stroke/TIA
Refractory migraines	Livedo reticularis
Less frequent (10-20% of cases):	
Cardiac valve disease	Preeclampsia or eclampsia
Premature birth	Hemolytic anemia
Coronary artery disease	
Uncommon (< 10% of cases):	
Epilepsy	Vascular dementia
Chorea	Thrombosis of the retinal vein or artery
Amaurosis fugax	Pulmonary hypertension
Leg ulcers	Digital gangrene
Osteonecrosis	Renal microangiopathy
Mesenteric ischemia	
Rare (< 1% of cases):	
Suprarenal hemorrhage	Transverse myelitis
Budd-Chiari Syndrome	CAPS

TIA: transitory ischemic attack; CAPS: Catastrophic APS. Adapted from Ruiz-Irastorza et al.^9^

Rarely, a devastating condition with rapid onset known as catastrophic antiphospholipid syndrome (CAPS) may be observed. This is characterized by multiple vascular occlusions (in more than three organs or systems) over a short period of time (less than 1 week). More than half of CAPS cases occur in the presence of identifiable trigger factors, such as bacterial or viral infections, surgical procedures, withdrawal of anticoagulant treatment, obstetric complications, neoplasms, or concomitant SLE. Catastrophic antiphospholipid syndrome is linked with elevated mortality rates, varying from 36.8% to over 50% of cases, depending on the publication.^10^^,^ 11

## DIAGNOSIS

The preliminary classification criteria, which were published in 1999 (Sapporo) and updated in 2006 (Sydney),^12^ define APS as the presence of at least one clinical criterion and one laboratory test criterion (listed in Table 2). The objective of these criteria is to define the syndrome’s characteristics for the purposes of etiologic and therapeutic studies. They are also frequently used for precise diagnosis of APS in clinical practice, but should not be considered absolute prerequisites for prescribing or withholding treatment.

**Table 2:** Updated criteria (Sydney) for classification of APS.

Clinical criteria	1. Venous, arterial, or small vessel thrombosis* confirmed objectively (imaging or histopathology**)
	2. Gestational morbidity
	(a) one or more unexplained occurrence of death of a morphologically normal fetus at or after the 10th week of gestation, confirmed by ultrasound or examination of the fetus; or
	(b) one or more premature deliveries (before the 34th week of gestation) of a morphologically normal neonate, caused by eclampsia, severe pre-eclampsia or placental insufficiency; or
	(c) three or more consecutive unexplained spontaneous abortions before the 10^th^ week of pregnancy, after ruling out maternal anatomic or hormonal causes and paternal and maternal chromosomal causes.
Laboratory criteria	(a) lupus anticoagulant (LA) detected according to ISTH§ guidelines on two or more separate occasions at least 12 weeks apart; or
	(b) anticardiolipin antibody (aCL) IgG or IgM detected in serum or plasma with medium to high titers (> 40 GPL or MPL units or > 99^th^ percentile) on two or more separate occasions at least 12 weeks apart, measured using standardized ELISA; or
	(c) anti-β2-glycoprotein I antibodies (anti-β2-GPI) detected in serum or plasma on two or more separate occasions at least 12 weeks apart, measured using standardized ELISA

* Does not include superficial venous thrombosis;

** Without significant inflammation of the vascular wall (vasculitis);

§ International Society on Thrombosis and Haemostasis. Adapted from Miyakis et al.^12^

Only laboratory test results and clinical manifestations that are most specific to APS were included in the classification criteria. Other clinical and laboratory findings that are also associated with APS are denominated “non-criterion” manifestations or tests. Non-criterion manifestations of APS include cardiac valve disease, livedo reticularis, skin ulcers, thrombocytopenia, nephropathy of APS, and neurological manifestations related to aPL.^12^^,^ 13 Non-criterion Laboratory tests include the antibodies aCL IgA, anti-β2-GPI IgA, anti-phosphatidylserine (aPS), anti-phosphatidylethanolamine (aPE), anti-prothrombin alone (aPT-A), anti-phosphatidylserine-prothrombin complex (aPS/PT), and anti-domain I of β2-glycoprotein I.^12^^,^ 14

### Effect of anticoagulant therapy on laboratory investigation of APS

In practice, it can sometimes be necessary to conduct laboratory investigations of APS for patients who are already being treated with parenteral or oral anticoagulant drugs. In this situation it is not expected that there will be a significant influence on the results of ELISA aCL and anti-β2-GPI tests, but there can be difficulties with correctly diagnosing LA.

Guidelines published by the International Society on Thrombosis and Haemostasis (ISTH)^15^ recommend that LA should be tested 1 to 2 weeks after withdrawal of treatment with vitamin K antagonists (VKA) or when the international normalized ratio (INR) value is less than 1.5. Treatment can be bridged using low molecular weight heparin (LMWH) when VKA are withdrawn, taking care to be sure that the last administration of heparin occurred more than 12 hours before the sample for LA testing is collected. Another possibility is to test for LA during treatment with VKA, at a point at which INR is between 1.5 and 3.0, diluting the patient’s plasma sample at a proportion of 1:1 with pooled normal plasma. In this case, low levels of LA may not be detected, since the sample is diluted by half.

One point that should be stressed is that the growing popularity of the new direct oral anticoagulants (DOAs), such as rivaroxaban and dabigatran etexilate, for treatment of patients with venous thromboembolism can lead to false-positive results in the usual tests for LA (dilute Russell's viper venom time [dRVVT] and activated partial thromboplastin time [APTT]) caused by therapeutic concentrations of these drugs.^16^^,^ 17 Specifically in the case of rivaroxaban, this effect can be avoided by using Taipan snake venom time or ecarin clotting time tests to diagnose LA,^18^ or by waiting at least 24 hours after the last dose of this medication before testing for LA using the usual techniques.^19^

## PATHOPHYSIOLOGY OF APS

Presence of aPL, and especially presence of LA mediated by anti-β2-GPI antibodies, confers an increased risk of thrombosis and/or gestational morbidity. Experimental infusion of autoantibodies from patients with APS potentiated formation of thrombi in mice with induced vascular injuries. However, the same effect was not seen in the absence of prior vascular injury, suggesting that APS occurs over two distinct phases (the double-hit hypothesis): (1) existence of a latent prothrombotic state, induced by circulating autoantibodies; (2) endothelial injury or activation, triggering the thrombotic event. Recent studies indicating elevated plasma levels of circulating microparticles derived from platelets and endothelial cells in patients with APS or aPL provide confirmation of the concept of a continuous prothrombotic state in these patients.^20^^,^ 21

Mechanisms potentially involved in the pathogenesis of APS include^22^:

- Increased immunogenicity of domain I of plasma β2-glycoprotein I, caused by oxidative stress;
- Conformational change to the β2-glycoprotein I molecule when it binds to cell membrane anionic phospholipids, enabling the binding of autoantibodies and the subsequent pathogenic effects;
- Interference of these autoantibodies with the nitric oxide endothelial synthase function;
- Increased expression of thromboplastin in endothelial cells and monocytes, induced by aPL;
- Uncontrolled activation of factor XI and consequent predisposition towards venous and arterial thrombosis;
- Potentiation of platelet activation by interaction between β2-glycoprotein I, platelet receptors, and anti-β2-GPI antibodies;
- Interference by antigen/antibody β2-GPI/anti-β2-GPI complexes in the protective function of annexin A5 against activation of coagulation;
- Antibody-mediated activation of the complement (C3 and C5), resulting in fetal loss and/or thrombosis;
- Increased expression of toll-like receptors 7 and 8 (TLR7 and TLR8); and
- In patients with APS nephropathy, aPL activation of the mammalian target of rapamycin complex (mTORC) pathway, inducing accentuated intimal hyperplasia and vascular disease.^23^

## TREATING APS – GESTATIONAL MORBIDITY

Treatment for patients with obstetric APS includes non-pharmacological measures such as management by a multidisciplinary team at a high-risk pregnancy clinic, rigorous clinical and laboratory control, and ultrasound monitoring of fetal growth and uteroplacental circulation.^24^^,^ 25 Patients with a history of APS with exclusively obstetric manifestations can be treated, in the event of future pregnancies, with low-dose aspirin (LDA) (75-100 mg/day) plus unfractionated heparin (UFH) or LMWH at prophylactic doses until 6 weeks after delivery.^24^ However, pregnant women with APS and a history of thrombotic events need antithrombotic therapy throughout the entire pregnancy and immediate postpartum period, with a combination of LDA and full anticoagulant doses of heparin (UFH or LMWH).^24^^,^ 26

Once the pregnancy-puerperium cycle is complete, patients who have exhibited gestational APS remain at increased risk of thrombotic events, even when there is no prior history of thrombosis, and in these circumstances indefinite primary prophylaxis with LDA is recommended, in addition to controlling the classic risk factors for thrombosis.^26^

## TREATING APS – THROMBOTIC MANIFESTATIONS

### Stratification of risk of thrombosis and hemorrhage

All treatment decisions for patients with APS should take account of the risk of thrombosis recurrence and risks resulting from the anticoagulant treatment (for example, hemorrhage).^27^ In general, it is recommended that risk of thrombosis should be stratified by identification of cardiovascular risk factors (primarily: smoking, hypertension, diabetes, dyslipidemia, and obesity), presence or absence of associated autoimmune diseases (for example, SLE), and definition of whether or not there is a high-risk aPL profile,^27^ which includes (1) positive LA result; (2) triple positivity, (i.e. LA, aCL, and anti-b2-GPI all positive in the same patient); and (3) persistently high aCL levels in patients with SLE. Factors that can increase the risk of hemorrhagic complications from anticoagulant treatment include concomitant use of aspirin (especially if > 100 mg/day), age > 75 years, prior severe hemorrhage, polypharmacy, cancer, and cerebral white matter abnormalities (leukoaraiosis).^27^

### Secondary thromboprophylaxis

Patients who have had one or more thrombotic events and meet the classification criteria for APS are at increased risk of recurrence of this type of event and long-term anticoagulant therapy is therefore indicated. In cases with a prior history of venous thrombosis, VKA are indicated with a target INR of 2.0 to 3.0. For patients with APS who have had arterial thrombosis, the prevalent recommendation,^1^^,^ 26 ^,^ 27 based on specialists’ opinions, is long-term treatment with VKA with a target INR of >3.0 (intensive regime), or INR of 2.0 to 3.0 in combination with platelet antiaggregation (LDA). Treatment for patients who have had thrombotic events but do not meet the classification criteria for APS, i.e. have positive results, but at low levels, and/or in whom aPL is not persistent, should be treated in the same way as if these tests had been negative.^27^

In cases of APS confirmed by the classification criteria (Table 2) and with arterial or venous thrombosis, the duration of secondary thromboprophylaxis is indefinite. However, for patients who have a first venous thrombosis event with an identified transient trigger factor and a low risk aPL profile (isolated, non-persistent positive result with low aCL or anti-β2-GPI titers – i.e. insufficient criteria to confirm APS), withdrawing anticoagulant therapy can be considered after 3 to 6 months, possibly supporting this decision with Doppler ultrasonography examination and D-dimer testing in order to rule out significant residual venous thrombosis.

### Primary thromboprophylaxis

Long-term primary thromboprophylaxis in patients positive for aPL but with no previous thrombosis is a controversial subject. A prospective study did not detect any benefit from using LDA,^28^ but a meta-analysis of 11 studies did detect a protective action from this intervention against thrombotic events (arterial, but not venous), and although this protection was eliminated when the analysis was restricted to prospective and higher-quality studies,^1^^,^ 29 another meta-analysis that used individual patient data from five cohorts also found evidence of protection.^30^

The following recommendations made by a task-force that was convened during the 13^th^ International Congress on Antiphospholipid Antibodies^27^ remain valid for management of patients with aPL but without antecedents of thrombosis: (1) assessment and control of associated cardiovascular risk factors; (2) use of LMWH or equivalent during periods of increased risk of thromboembolic events (surgery, immobilization, puerperium); (3) prescription of an antiplatelet drug (LDA) for higher-risk patients, such as patients with concomitant prothrombotic risk factors and patients with a high-risk aPL profile (see above); and (4) use of an antiplatelet drug (LDA) and hydroxychloroquine for patients with SLE who are positive for LA or have persistently positive high-level aCL test results.

### Direct oral anticoagulants, non-vitamin K antagonists

Secondary, long-term thromboprophylaxis with warfarin or other VKA involves several inconvenient aspects, such as the need for regular monitoring of INR, multiple drug and dietary interactions and, in some cases, difficulty with achieving or maintaining adequate and stable levels of anticoagulation. Another problem mentioned in the literature is the variable interaction between LA and the reagents used to evaluate prothrombin time (PT), which sometimes results in prolonged baseline values and reduced reliability of INR values for anticoagulation intensity monitoring.^16^^,^ 31

Direct oral anticoagulants, including a direct thrombin inhibitor (dabigatran etexilate), and two direct factor Xa inhibitors (rivaroxaban and apixaban) are commercially available in Brazil, and have been approved for, among other indications, DVT and/or venous thromboembolism prophylaxis. The efficacy and safety of their use for these indications have been confirmed in at least three systematic reviews.^32^^-^ 34 The advantages offered by these medications over VKA are as follows: administration in fixed doses, usually without the need for laboratory monitoring of their anticoagulant action; absence of significant interactions with alcohol or food; and fewer significant drug interactions.^16^^,^ 17

In view of the inconveniences related to use of VKA and the advantages offered by DOAs, it is natural that there is interest in employing the newer drugs to treat APS as well. It has been estimated that around 10% of patients with DVT have APS,^35^ from which it can be deduced that there were probably patients with APS enrolled on studies of DOAs. However, considering that these studies did not systematically document aPL status, their results cannot be generalized to patients with APS.^36^ Furthermore, the intervention with which DOAs were compared in those studies was warfarin with a target INR of 2.5 (2.0 – 3.0), which is generally appropriate for secondary prophylaxis of venous thrombosis, but may offer an insufficient level of protection for patients with APS, particularly those with arterial thrombosis. There are currently, to the knowledge of the authors of the present article, at least two clinical trials underway assessing rivaroxaban for patients with APS: the Rivaroxaban In Antiphospholipid Syndrome (RAPS) study,^36^ which has been recruiting patients with APS and prior history of venous thrombosis, and the Rivaroxaban in Thrombotic Antiphospholipid Syndrome (TRAPS) study,^37^ in which the study population comprises patients with APS and venous, arterial or microvascular thrombosis, and a triple-positive aPL profile. When the results of these studies become available, they may elucidate the role that DOAs have to play in treatment of patients with APS.

While the results of specific clinical trials are not yet available, there are several reports in the literature of cases and/or case series in which DOAs were used on patients with APS, with contradictory results. In a series^16^ of 35 patients with APS and history of venous thrombosis with indication for moderate intensity anticoagulation (INR 2 to 3), but in whom there were difficulties achieving or maintaining the therapeutic target, VKA were substituted with 20 mg rivaroxaban per day, after which no new venous thromboembolism events were observed during follow-up with a median duration of 10 (6-24) months. Another publication^38^ describes treatment of 26 patients with APS and history of venous and/or arterial thrombosis with dabigatran or rivaroxaban. After median follow-up of 19 (8-29) months, treatment was discontinued in just four cases (recurrent thrombosis in one patient, hemorrhagic complications in two patients, and recurrent migraines in one patient). However, we also found reports in the literature describing highly unfavorable experiences with use of DOAs in patients with APS. Schaefer et al.^39^ reported three cases (two of primary APS with arterial events and one of APS and SLE with venous events) that exhibited further thrombotic events after warfarin was substituted with a DOA (dabigatran or rivaroxaban). Win and Rodgers also described three cases of difficult to treat APS that exhibited further thrombotic or ischemic events while on rivaroxaban or dabigatran.^40^ In another case series, reported by Signorelli et al.,^41^ eight patients with APS and prior histories of venous and/or arterial thromboses (three of whom were triple positive for aPL) had additional thrombotic events while on treatment with rivaroxaban; seven were put back onto treatment with VKA and did not have further thrombosis relapses thereafter.

A task force on antiphospholipid syndrome treatment trends met during the 14th International Congress on Antiphospholipid Antibodies^42^ and their recommendations state that VKA are still the foundation of anticoagulation in patients with thrombotic APS, although DOAs can be considered in patients with recurrent or initial venous thromboembolism that occurs in the absence of or with subtherapeutic levels of anticoagulation and only if there is known allergy to or intolerance of VKA or inadequate anticoagulant control. According to the same recommendations, there are no data to support use of DOAs in patients with APS and recurrent venous thromboembolism that occurs despite therapeutic anticoagulation levels or who exhibit arterial thrombosis caused by APS. Additionally, these recommendations highlight the crucial role of patient compliance with DOA treatment, since the anticoagulant effect is normally not monitored, with the consequence that poor patient compliance with VKA treatment is not a reason for prescribing DOAs. Finally, they also caution that there are no antidotes for DOAs that could be used in the event of severe hemorrhagic complications or urgent need for surgery.

A summary of the treatment of APS according to the type of manifestation (gestational or thrombotic) can be seen in Table 3. The primary and secondary thromboprophylaxis in APS are summarized in Table 4.

**Table 3:** Summary of treatment for APS.

APS with gestational morbidity	
General measures	Multidisciplinary team. Clinical/laboratory/ultrasound monitoring
Pharmacotherapy	No prior thrombosis: LDA + prophylactic dose of UFH or LMWH With prior thrombosis: LDA + therapeutic dose of UFH or LMWH After delivery/puerperium: LDA
APS with thrombotic manifestations	
General measures	Risk stratification for thrombosis and/or hemorrhage
Pharmacotherapy	Secondary thromboprophylaxis

**Table 4:** Thromboprophylaxis in APS.

Secondary thromboprophylaxis (confirmed APS)	
Venous thrombosis	Anticoagulation with VKA, INR 2.0 to 3.0
Arterial thrombosis	Anticoagulation with VKA, INR > 3.0 or Anticoagulation with VKA, INR 2.0 to 3.0 + LDA
Primary thromboprophylaxis (positive for aPL, clinical criteria not present)	
General	Thromboprophylaxis with LMWH or equivalent during high-risk periods (surgery, immobilization, puerperium)
With associated risk factors (cardiovascular risk, high-risk aPL profile)	LDA
Patients with SLE and positive for LA or high/persistent aCL levels	LDA + hydroxychloroquine

### Treatment of catastrophic APS (CAPS)

The severity of CAPS demands immediate and vigorous action. Reversible precipitating factors (for example, infections) should be identified and treated. Analyses of case series and case reports^10^^,^ 11 ^,^ 43 ^-^ 45 indicates that survival rates are higher with combined triple therapy with full anticoagulation with heparin and corticoid therapy in high doses combined with plasmapheresis and/or intravenous immunoglobulin (IgIV), preferring the latter if there is an active infection. If both are used (plasmapheresis and IgIV), the plasmapheresis sequence should be concluded before starting administration of IgIV. If SLE or other underlying autoimmune disease are present in addition to CAPS, additional treatment with cyclophosphamide or another immunosuppressant may be indicated. Use of rituximab has been suggested for treatment of recurrent refractory CAPS, when there is microangiopathic hemolytic anemia or when anticoagulation is contraindicated because of concomitant hemorrhagic complications.^11^

### Perioperative management of patients with aPL or APS^46^

Surgical procedures can trigger thrombotic events^47^ and even CAPS^11^ in patients with aPL or APS and adequate preoperative planning of prophylaxis is therefore necessary. The medical team (clinical team, surgeon, anesthetist) should, by consensus, define the approach that will be taken in terms of the timing of preoperative suspension of VKA, using LMWH as a treatment bridge, application of intermittent compression devices, early postoperative mobilization, postoperative reintroduction of VKA, and laboratory monitoring strategy.^46^ This approach should take into consideration any history of thrombotic or hemorrhagic events, whether a clinically relevant aPL profile exists (titers > 40 GPL or MPL units, persistence > 12 weeks, and detection of LA according to the ISTH guidelines) and presence of other risk factors for thrombosis or hemorrhage.^46^^,^ 48 Although 20 to 40% of patients with APS have thrombocytopenia, most often above 70,000 platelets/mm^3^, this rarely results in significant hemorrhage and does not reduce the risk of thrombosis.^49^ In the absence of anticoagulant treatment, an elevated APTT (above the reference value) may indicate presence of LA and a minimally prolonged PT (above the upper limit of normality) may indicate presence of antiprothrombin antibodies. More accentuated PT abnormalities (INR > 2.0) require the investigation of other causes including the LA-associated hypoprothrombinemia.^46^^,^ 50

Prevention of perioperative thrombosis in patients with APS includes general measures, such as (i) minimizing intravascular manipulation for access and monitoring; (ii) reducing venous stasis, avoiding use of tourniquets for drawing blood samples, and reducing the frequency of inflation of cuffs to take blood pressure; (iii) thromboprophylaxis, combining mechanical modalities (graduated compression stockings or intermittent pneumatic compression devices) and pharmacological measures.^46^ Perioperative pharmacological thromboprophylaxis in patients with APS on oral anticoagulation with VKA is provided parenterally using LMWH (enoxaparin) or UFH to provide a therapeutic bridge, returning to VKA as soon as possible postoperatively (UFH or LMWH should only be withdrawn when INR is on target).^46^^,^ 51 Occurrence of hemorrhagic complications does not eliminate the risk of thromboembolism, and in these situations intensive use of mechanical methods is recommended and, as soon as possible, resumption of pharmacological methods of thromboprophylaxis.^46^

## FINAL COMMENTS

Antiphospholipid Syndrome is very important in vascular medicine because it is a recognized cause of recurrent venous and/or arterial thrombosis. Diagnosis is dependent on recognition of its clinical thrombotic and/or gestational manifestations and on requesting and interpreting the appropriate laboratory tests for the syndrome. The results of tests for LA can be affected by use of parenteral or oral anticoagulant medications and it is recommended that particular care is taken with choosing the time of sample collection and with interpreting the results in these circumstances. Increasingly, additional pathogenic APS mechanisms are being revealed, which could facilitate development of new treatment options that do not involve anticoagulants. An appropriate approach to treatment of these patients should take account of their history of thrombotic events and/or prior gestational morbidity and other associated cardiovascular risk factors. Although DOAs offer several benefits over use of warfarin or other VKA for treatment and prophylaxis of venous thrombosis in the general population, confirmation of their efficacy for APS is still awaiting the results of specific trials. Perioperative management of patients with APS demands special precautions related to prophylaxis against both thromboembolic manifestations and hemorrhagic complications.
